# Opportunities and challenges of glioma organoids

**DOI:** 10.1186/s12964-021-00777-0

**Published:** 2021-10-11

**Authors:** Xiangdong Xu, Lingfei Li, Linting Luo, Lingling Shu, Xiaoli Si, Zhenzhen Chen, Wenqing Xia, Jinyu Huang, Yang Liu, Anwen Shao, Yiquan Ke

**Affiliations:** 1 The National Key Clinical Specialty, The Engineering Technology Research Center of Education Ministry of China, Guangdong Provincial Key Laboratory On Brain Function Repair and Regeneration, Department of Neurosurgery, Zhujiang Hospital, Southern Medical University, Guangzhou, 510282 People’s Republic of China; 2 Department of Neurology, Affiliated Hangzhou First People’s Hospital, Zhejiang University School of Medicine, Hangzhou, People’s Republic of China; 3 Department of Neurology, Liwan Central Hospital of GuangZhou, Guangzhou, People’s Republic of China; 4 State Key Laboratory of Oncology in South China, Collaborative Innovation Center for Cancer Medicine, Sun Yat-Sen University Cancer Center, Guangzhou, 510060 People’s Republic of China; 5 Department of Hematological Oncology, Sun Yat-Sen University Cancer Center, Guangzhou, 510060 People’s Republic of China; 6 Key Laboratory of Pharmaceutical Biotechnology, The University of Hong Kong, Hong Kong, People’s Republic of China; 7 Department of Neurology, the Second Affiliated Hospital, School of Medicine, Zhejiang University, Hangzhou, People’s Republic of China; 8 Department of Hematology, Affiliated Hangzhou First People’s Hospital, Zhejiang University School of Medicine, Hangzhou, People’s Republic of China; 9 Department of Cardiology, Affiliated Hangzhou First People’s Hospital, Zhejiang University School of Medicine, Hangzhou, People’s Republic of China; 10 Department of Neurosurgery, the Second Affiliated Hospital, School of Medicine, Zhejiang University, Hangzhou, 310009 People’s Republic of China

**Keywords:** Glioma, Glioblastoma, Organoids, Tumor microenvironment, Cancer stem cells, Endothelial cells

## Abstract

Glioma is the most common primary brain tumor and its prognosis is poor. Despite surgical removal, glioma is still prone to recurrence because it grows rapidly in the brain, is resistant to chemotherapy, and is highly aggressive. Therefore, there is an urgent need for a platform to study the cell dynamics of gliomas in order to discover the characteristics of the disease and develop more effective treatments. Although 2D cell models and animal models in previous studies have provided great help for our research, they also have many defects. Recently, scientific researchers have constructed a 3D structure called Organoids, which is similar to the structure of human tissues and organs. Organoids can perfectly compensate for the shortcomings of previous glioma models and are currently the most suitable research platform for glioma research. Therefore, we review the three methods currently used to establish glioma organoids. And introduced how they play a role in the diagnosis and treatment of glioma. Finally, we also summarized the current bottlenecks and difficulties encountered by glioma organoids, and the current efforts to solve these difficulties.

**Video Abstract**

**Supplementary Information:**

The online version contains supplementary material available at 10.1186/s12964-021-00777-0.

## Background

Gliomas are the most frequent primary brain tumor. According to the World Health Organisation (WHO) classification, gliomas are divided into well-differentiated low-grade astrocytomas (WHO I–II), anaplastic astrocytomas (WHO III), and glioblastoma multiforme (GBM, WHO IV) [1]. GBM is the most aggressive type of glioma. It accounts for 14.6% of adult primary brain and other central nervous system (CNS) tumors, 48.3% of primary malignant brain tumors and 57.3% of all gliomas [2]. The prognosis of GBM is very poor [3]. Despite surgical resection, GBM is easy to relapse because of its rapid growth in the brain, resistance to chemotherapy and high invasiveness. The median survival time of GBM is about 15 months, and there is no cure at present. As a result, the 5-year survival rate is still less than 10% [4, 5].

Tumors are a complex system. In the process of initiation, maintenance and development, their different components are dynamically and continuously regulated. Gliomas, and particularly GBM, are some of the most comprehensively characterized cancers. Great efforts have been made to overcome the treatment platform that exists after the current standard or even experimental therapy. Unfortunately, despite all these efforts, no significant progress has been made in the way we treat patients, and the cure is still a long way from what we have achieved so far. Therefore, there is an urgent need for a platform to study the cytodynamics of GBM in order to find the characteristics of the disease and develop more effective treatments.

## Defects in previous models

Before the emergence of glioma organoids, scientific researchers had developed a variety of models for the study of gliomas, such as the following 2D models and animal models. Although these models provide great help for our research, they also have many defects.

### 2D models

Historically, cancer cell lines have been an easy-to-operate model for the study of tumor molecular biology and drug screening. In the past few years, many GBM immortalized cell lines, including U87, U251 and T98G, have been established to study the mechanisms related to GBM biology [6]. However, after many passages in the standard medium containing serum, the human GBM cell line showed a large number of genotypic and transcriptome changes, which completely disappeared the similarity with the primary tumor [7, 8]. In addition, when transplanted into nude mice, human GBM cell lines are usually more homogeneous than their source tumors, showing limited necrosis and microvascular changes [9].To sum up, these characteristics make GBM cell line a defective model for studying the occurrence and development of GBM [10, 11].

The shortcomings of 2D cell culture methods are summarized as follows: (1) lack of interaction between glioma cells and tumor microenvironment (TME) [12, 13], (2) lack of oxygen, nutrients and pH gradients [14, 15], (3) lack of physiological inputs from other metabolically active organs (such as liver, kidney, etc.), and (4) genomic changes after long-term culture [12, 16]. Therefore, simple 2D cell line culture experiments are becoming less and less convincing.

### Glioma stem cells

In many tumor tissues, a small number of cells have the ability to self-renew and proliferate indefinitely, and have the potential for multidirectional differentiation. They have the basic characteristics of stem cells and are called cancer stem cells (CSCs). With further research on malignant gliomas, the researchers also successfully isolated glioma stem cells (GSCs) from glioma tissues and discovered a series of characteristics similar to neural stem cells [17]. GSCs theory not only deepens people's understanding of the origin of malignant glioma, but also provides new ideas for the study of its occurrence and development mechanism, and provides a new direction for the treatment of the disease [18–20]. The original GSCs cultures in serum free media are performed in 3D. When cultured under serum-free conditions supplemented with epidermal growth factor (EGF) and basic fibroblast growth factor (bFGF), GSCs can self-renew to produce spheres called "glioma neurospheres" [21, 22]. In some literature, these cultures are also referred to as GBM neurospheres, brain tumor-initiating cells or glioma stem-like cells. GSCs were shown to better preserve the genetic background of tumors, to maintain a certain degree of phenotypic heterogeneity and molecular gradients [7, 23, 24]. At the same time, because of the 3D structure of the sphere, it can well simulate the oxygen and nutrient gradients of tumors in the body. Secondly, because glioma neurospheres can be suspended in conventional specific stem cell culture medium or soaked in gel, they can be used as an important tool for high-throughput drug screening [25]. Although GSCs models have many advantages over 2D cell line models, GSCs are not perfect. Because GSCs do not retain the complex structure of the tissue structure including extracellular matrix (ECM) and tumor microenvironment (TME). Since GSCs are generally maintained as long-term cultures, they also suffer to some extent from clonal selection and genetic drift [26].

In addition, more and more evidences indicate that CSCs may not constitute a clear cell entity, but a cell state that adapts to microenvironmental cues, thus challenging the CSCs model [27]. Initial research on GBM showed that only CSCs-marked positive cells can form tumors [21, 28]. Later research reports showed that GSCs and glioma cells either have no difference in tumorigenic potential [29–31], or both parts are tumorigenic but have different potency [32–34]. At the same time, some studies have shown that positive GSCs can be derived from the negative parts, and regain the initial heterogeneity, support strong tumor plasticity, and reconstruct the phenotypic heterogeneity within the tumor [30, 32, 34]. A large number of data supporting the concept of plasticity indicate the role of microenvironment in the formation of spatial and temporal heterogeneity phenotypes [35–37]. Interestingly, recent data further indicate that GBM CSCs alone have limited tumorigenic potential, and crosstalk with tumor cells representing more differentiated phenotypes creates a supportive niche and promotes tumor growth [13, 17]. These results indicate that tumor cell plasticity and intratumor phenotypic heterogeneity play a key role in shaping tumor progression. In summary, the simple GSCs model cannot restore the full picture of glioma. Therefore, it is necessary to develop a new generation of glioma model to more truly restore the situation of glioma in vivo.

### In vivo mouse models

Considering the complex relationship between environmental influences and cell–cell interactions in the brain, in vivo small animal models have been established to study the mechanism of GBM development. The two most classical models are patient-derived xenografts (PDXs) and genetically engineered mouse models (GEMMs). Although these two classic animal models provide great help for the study of glioma, they still have some defects.

Although the PDXs model retains the key molecular and histological characteristics of human glioblastoma, it is not suitable for studying tumor occurrence. And the PDXs model is limited by the inherent differences between human and mouse brain cells, as well as the variability in tumor latencies, and real-time experimental operations [38]. Moreover, the long process of making PDXs model, the high cost and the existence of certain moral and ethical problems greatly reduce its practicability.Most importantly, mouse PDXs lacks human tumor microenvironment, which is the limiting factor for modeling human GBM [39].

As a supplement to the PDXs model, GEMMs can be used to evaluate tumor progression in microenvironments similar to endogenous cancer onset conditions. Different from animal models of cancer cell inoculation, GEMMs are tumor models developed in the microenvironment of natural immune maturity. The histopathological and molecular characteristics of tumors that appear in advanced GEMMs are very similar to their human counterparts, show genetic heterogeneity, and can spontaneously develop into metastatic disease. Therefore, GEMMs are usually superior to cancer cell vaccination models, which show no or limited heterogeneity and usually metastasize from the beginning. GEMMs have been successfully used to validate candidate cancer genes and drug targets, evaluate treatment effects, analyze the impact of tumor microenvironment, and evaluate drug resistance mechanisms.

However, because GEMM is an expensive and time-consuming model, its application is limited [10]. Secondly, the inherent differences between human and rodent brain characteristics may also lead to misleading interpretation of the experimental results [40]. For example, LiuSJ and his colleagues hope to simulate the mechanism of lncGRS-1 in the human body through a mouse model. While lncGRS-1 is primateconserved, this lncRNA does not exist in rodents, making traditional in vivo mouse models of glioma suboptimal for assessing potential toxicity of lncGRS-1 knockdown in normal brain tissue [41]. Therefore, GEMMs are not the best platform to resemble human tumor heterogeneity. In general, it is clear that the in vitro model needs to be improved to better represent the biological characteristics and therapeutic response of gliomas.

## A brief overview of human cerebral organoids

**Fig. 1:**
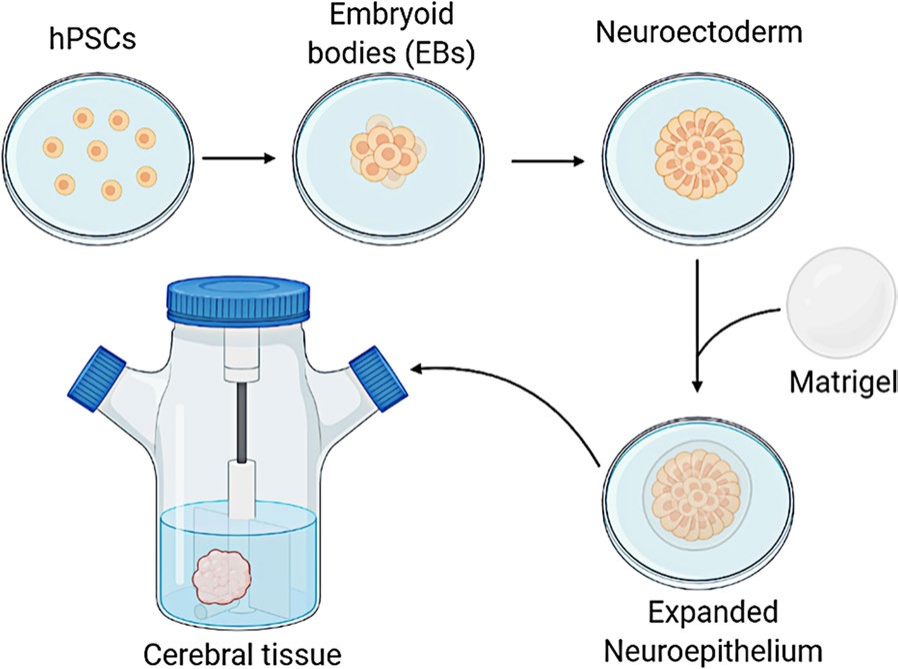
Schematic of cerebral organoid culture. First, pluripotent stem cells (PSCs) were cultured as embryoid bodies (EBs), and then differentiated into neuroectoderm. Neuroectodermal tissues were then maintained in 3D culture and embedded in droplets of Matrigel to provide a scaffold for more complex tissue growth. These Matrigel droplets were then transferred to a spinning bioreactor to enhance nutrient absorption. Adapted from reference Lancaster et al. [43]. Created with BioRender.com

Recently, scientific researchers have constructed a 3D structure called Organoids, which is similar to the structure of human tissues and organs. Organoids is a 3D structure usually formed by embedding patient-derived stem cells into the Matrigel matrix and cultured with a series of growth factors. These cells proliferate and differentiate within a few days, self-organizing in an organic structure [42]. In 2013, Lancaster and colleagues generated a robust protocol for the derivation of cerebral organoids. Starting from induced pluripotent stem cells (iPSC) cultured into embryoid bodies, they induce differentiation into neuroectoderm and embed the cells in MatrigelTM droplets. These droplets are then cultured in differentiation medium containing EGF/FGF2 and transferred to a rotating bioreactor (Fig. 1) [43]. Human cerebral organoids are an emerging technology currently under development, which has attracted widespread attention in the scientific community and the public domain. These in vitro constructs use the self-organizing properties of iPSC to summarize the key steps in the process of neural development, so that the neural tissue is very similar to the human brain [44].

It is worth noting that cerebral organoids summarize the early stages of human brain development [45, 46]. Neurons have a cone-shaped feature when they mature, with moderate spatial separation, and more importantly, they exhibit a high degree of outer radial glial cell population [47]. For these reasons, cerebral organoids are widely used to summarize the developmental stages of nerve tissue and simulate neurodevelopmental disorders in vitro [48–50]. Recently, studies have shown that cerebral organoids exhibit good repeatability. Its organoid-to-organoid variability is comparable to that of a single endogenous brain and is consistent in the cell types produced [51]. Due to cerebral organoids good repeatability, this makes it a reliable platform for studying brain diseases.

Because the cerebral organoids can summarize some of the key characteristics of the human brain, including cell distribution and organization, physiological structure, electrical activity and neuronal network [43, 52, 53]. Therefore, the cerebral organoids has become a unique model for exploring the mechanisms of nervous system diseases.

## Cerebral organoids as a model for glioma

The appearance of the same cerebral organoids also provides a very good platform for glioma research. We call the cerebral organoids used for glioma research as glioma organoids. The current glioma organoids mainly have the following three types, which are now summarized as follows.

### Obtaining glioma organoids through gene editing of cerebral organoids

GBM is a highly heterogeneous brain cancer. Several genetic alterations have been described to be involved in the onset of the disease, including the amplification of epidermal growth factor receptor (EGFR) gene, mutations in isocitrate dehydrogenase (IDH), telomerase reverse transcriptase (TERT), phosphatase tensin homologue (PTEN), neurofibromatosis type 1 (NF1), platelet-derived growth factor receptor alpha (PDGFRα), tumor protein p53 (TP53), retinoblastoma protein (RB), cyclin-dependent kinase inhibitor 2A (CDKN2A) and altered promoter methylation of O6-Methylguanine-DNA methyltransferase (MGMT) [54–56]. At the same time, in the in vitro model, the cerebral organoids derived from human tissue have the advantage of imitating the interaction between in vivo structure and environment, and have more reliable clinical significance compared with the mouse derived model. With these factors in mind, gene editing in human brain organoids has made it possible to study the early stages of tumorigenesis and cancer progression [57].

**Fig. 2:**
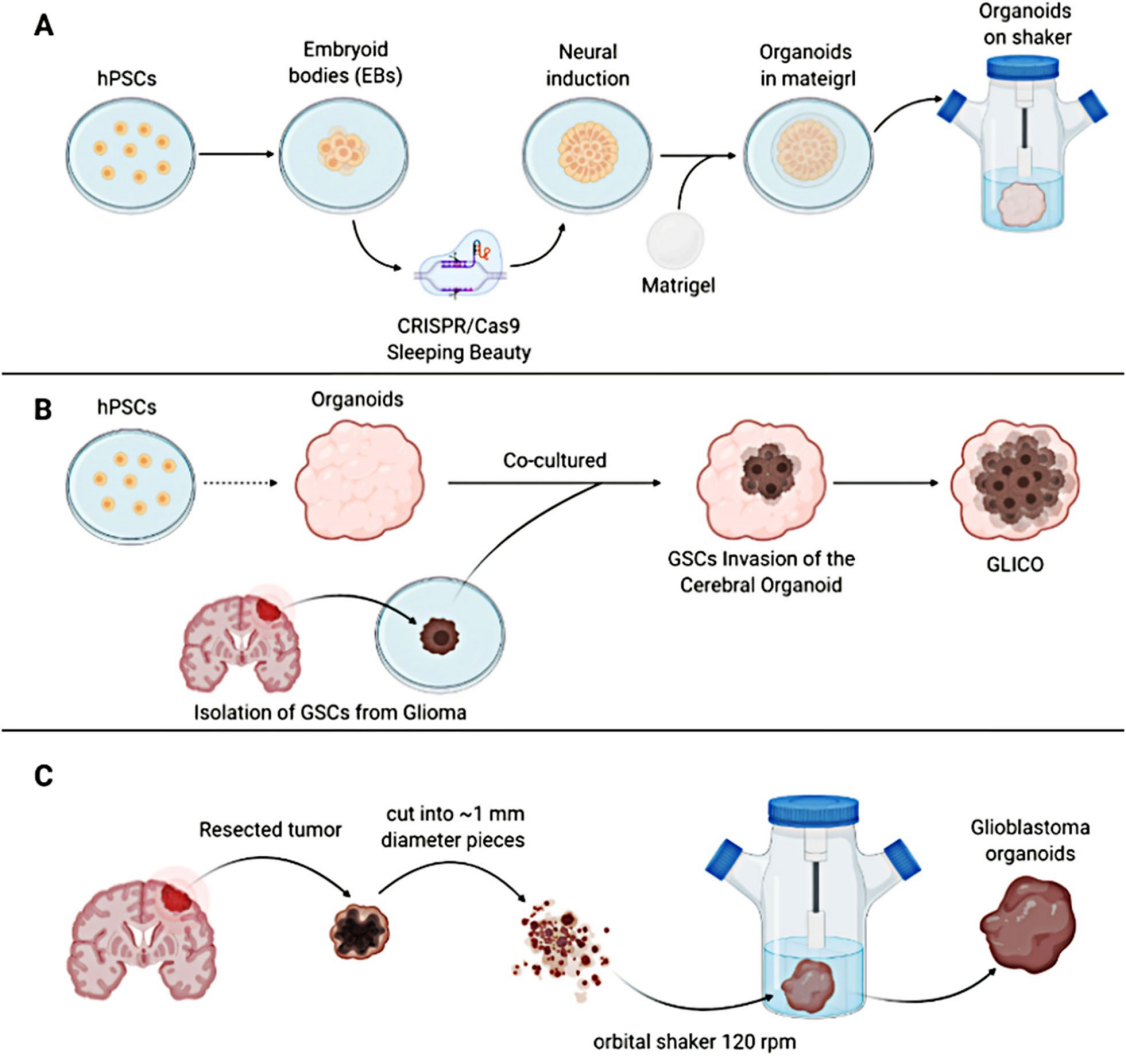
Glioma organoids. **a** Obtain glioma organoids through gene editing. CRISPR/Cas9 mutagenesis and Sleeping Beauty (SB) transposon-mediated gene insertion have served for this purpose by introducing clinically relevant oncogenic mutations into healthy human cerebral organoids in order to develop glioma tumors. **b** Glioma organoids were obtained by co-cultivation with GSCs. Cerebral organoids were obtained from 3D human embryonic stem cells or patient iPSCs, and then co-cultured with patient-derived GSCs. **c** Tumor derived glioma organoids. Cut the excised glioma tissue into pieces with a diameter of about 1 mm. The tumor pieces were cultured in the GBO medium on an orbital shaker for 1–2 weeks. Created with BioRender.com

In order to study the role of these mutant genes in the occurrence of gliomas, genomic engineering has been used to generate glioma tumor models in PSC-derived cerebral organoids. CRISPR/Cas9 mutagenesis and Sleeping Beauty (SB) transposon-mediated gene insertion have served for this purpose by introducing clinically relevant oncogenic mutations into healthy human cerebral organoids in order to develop glioma tumors (Fig. 2a) [40, 58]. At present, a variety of glioma organoids models have been established by genetic engineering. Bian and colleagues conducted a groundbreaking study in which they combined Sleeping Beauty transposon-mediated oncogene insertion with CRISPR/Cas9 mutations in tumor suppressor genes [40]. The authors created an in vitro 3D model called "neoplastic brain organs" (neoCOR), which enabled them to summarize some of the most common and clinically relevant combinations of functional mutations observed in GBM. Specifically, they generated three GBM models carrying the following mutations: CDKN2A−/CDKN2B−/EGFROE/EGFRvIIIOE, NF1−/PTEN−/TP53−, and EGFRvIIIOE/CDKN2A−/PTEN−. NeoCORs showed similar transcriptional spectrum to those observed in patients, and showed typical clinical markers related to GBM phenotype. NeoCORs also showed a variety of glial markers, such as S100 β and GFAP, which were positive for proliferation markers Ki67 and other tumor markers. Interestingly, GB-like neoCORs xenografted in immunocompromised mice can proliferate and produce tumor-like areas characterized by local tissue infiltration. At the same time, GB-like organoids were also shown to be suitable for drug screening [40].

Similarly, Ogawa and his colleagues used the methods described by Lancaster and Knoblich to grow human cerebral organoids and make them grow and mature for 4 months. At this time, the organoids have shown normal cortical structures and markers. At this time, CRISPR/CAS9 technology was used to mediate the homologous recombination of oncogene HRasG12V and TP53 tumor suppressor gene. This kind of genome insertion will not only destroy the tumor suppressor gene TP53, but also activate the oncogene HRasG12V, which encodes the expression of RAS protein. After two weeks of this process, the transfected cells could be observed by tdT and GFP signals. By the eighth week, almost 6% of the cells in the organoids are cancer cells. Therefore, this method can directly and continuously observe the occurrence of gliomas [58].

Genetic engineering of human cerebral organoids is a new technology, which has been proved to be able to generate in vitro glioma models combined with the most common clinical gene mutations. And the technique allows for the analysis of phenotypic and molecular consequences in a specific genetic context. Considering the difficulty of collecting samples from patients, especially in the early stages of GBM, gene editing of human cerebral organoids may open up a new field of vision for the treatment of gliomas. Using this technique, we can establish a reliable platform to study the occurrence and progress of GBM, to analyze important GBM markers, and to perform drug screening [10].

### Obtaining glioma organoids through co-cultivation of cerebral organoids and GSCs

The invasive ability of glioma cells depends on the complex interaction between tumor cells and the surrounding TME. These TME include microglia, bone marrow-derived macrophages, astrocytes, oligodendrocytes, neurons, glial and neuronal precursor cells, pericytes, endothelial cells and extracellular matrix (ECM) [59]. TME plays an important role in the occurrence and development of gliomas.

Leite and colleagues demonstrated that 3D co-culture of human GBM cell lines and microglia can support the growth and migration of glioblastoma, thus providing a protective environment for GBM [60]. They also show the new potential role of microglia in glioblastoma: microglia seem to regulate the drug resistance of tumor cells. Similarly, another study shows that astrocytes in TME are also involved in regulating drug resistance in gliomas. Moreover, the interaction between astrocytes and GBM cells may be related to the increase of tumor growth and invasion [61]. And it has been shown that direct contact between astrocytes and 3D co-culture of GBM cells can enhance the formation of glioblastoma [62]. These observations show that TME plays an important role in tumor tissue.

In order to simulate the complex interaction between glioma cells and the surrounding tumor microenvironment in vitro, several techniques have been developed. Among them, co-culture of GSCs with cerebral organoids is the most promising. Specifically, co-culture can well represent similar tissue models in humans and thus gain an in-depth understanding of the natural interaction between cell populations [63]. More deeply, compared with the 2D model, the cerebral organoids platform provides additional dimensions for cell proliferation and interaction, promoting the spatial organization of cell morphology and cell–cell or cell-extracellular matrix signal transduction.

Da Silva and colleagues developed a cerebral organoid model of glioma called GLICO. They show that glioma CSCs can infiltrate healthy cerebral organoid after 24 h of co-culture with healthy cerebral organoid of different ages [64]. They highlight how spherical co-cultures from GBM cells or neural progenitor cells infiltrate early cerebral organoid, leading to the formation of mixed organisms showing aggressive tumor phenotypes. Based on these findings, cerebral organoids were obtained from 3D human embryonic stem cells or patient iPSCs, and then co-cultured with patient-derived GSCs. The injected GSCs had the capability to penetrate in the cerebral organoids, forming tumors called “cerebral organoid glioma” (GLICO) (Fig. 2b) [65]. The GLICO model addresses many of the limitations of previous models because it allows people to study patient-specific GBM ex vivo in a microenvironment similar to the original human brain. The biological behavior and histopathological characteristics of patient-derived GBMs grown in brain organoids are closely related to the phenotype of surgical and autopsy specimens, which proves the clinical relevance of this model. In addition, the study also shows that the GLICO model maintains the key genetic characteristics and molecular signaling network of the parental tumor. In addition, because the model is cultured in vitro, it is suitable for experimental manipulation, drug therapy, precise control of physiological and environmental variables, and high-throughput drug screening [65]. In general, the co-culture of cerebral organoids provides an encouraging opportunity for the study of gliomas, allowing people to explore GBM biology and study the molecular mechanism of tumor invasion in the primitive human brain environment. At the same time, it also opens a new door for the treatment of gliomas.

### Obtaining glioma organoids from tumor material alone

The above organoids model provides the possibility to study normal tissue-tumor interaction. However, because there are only normal nerve cells and glioma cells, there is a lack of key elements of glioma cell components. In order to solve the above-mentioned problems, great efforts have been made to establish organoids derived from tumor materials. This kind of organoids retains the heterogeneity of parental tumor, relative 3D spatial tissue and basic interaction with ECM [66–68].

#### Obtaining glioma organoids from CSCs

In 2016, Jeremy Rich and his colleagues obtained glioblastoma organoids for the first time from finely minced tumor biopsies of both patients and genetically modified GB mice models [69].

Briefly, organoids were formed by suspending tumor cells in Matrigel and forming 20 µl pearls on parafilm molds prior to culture. Then transfer the newly formed organoids to a 10 cm or 6-well plate in a complete medium supplemented with EGF, bFGF, B27, glutamine, sodium pyruvate and antibiotics, and culture for 4 days without shaking. After 4 days, the organoids were transferred to the orbital shaker in the tissue culture incubator and cultured at 60–80 RPM to obtain mature glioma organoids. These GBM organs can be stably cultured for more than a year, and once implanted in situ, they can cause highly diffusive and invasive gliomas [69].

The advantage of such glioma organoids is that they can more effectively mimic the tumor microenvironment. They have been shown to produce gradients in stem cell density and hypoxia. Specifically, GSC near the surface of such organs often split and die, while GSC in hypoxic areas is static. The heterogeneity of cells and microenvironment in tumor organoids makes it possible to simultaneously cultivate stem cells and non-dry glioblastoma cell populations with different functions and phenotypes. Organoid culture can study dry and non-dry glioblastoma cell populations in the same culture, study the interaction between proliferation and CSC in hypoxic niches, and further analyze the subpopulation hierarchy of glioblastoma stem cells structure [69].

#### Obtaining glioma organoids from glioma tissue

In 2020, Jacob et al. used organoid technology to develop a glioma organoids derived from tumor tissue, which can preserve cellular structure and maintain different cell-to-cell interactions (Fig. 2c). They cultured the chopped tumor tissue in an organoid medium and placed it on an orbital oscillator to increase the spread of nutrition and oxygen. As a result, round organoid were formed by the end of the second week, many of which were able to retain their CD31 + vascular system and resemble hypoxic niches 300 µm far from these vessels. Through the identification of several histological markers, it was confirmed that they were similar to the strong cellular heterogeneity of parental tumors. Through single cell transcriptome analysis, the authors determined that both tumor and non-tumor cell populations (such as lymphocytes, macrophages and microglia) were preserved after 2 weeks of culture. And it showed strong invasiveness when orthotopic transplantation was performed in immunocompromised mice. Finally, on the basis of this technique, the author has established an organoid biological bank, which can be used to test different types of treatments in vitro [70]. Therefore, organoid derived from tumors can better summarize some of the details of TME, such as stem cell gradients and hypoxia.

## Application of glioma organoids

### Used for drug sensitivity test

As mentioned above, organoid can be derived from patients' tumor tissues, and many biological banks have been established, so they can become valuable tools for drug screening [65, 71]. Because the establishment of human glioma transplantation model in mice is time-consuming and relatively expensive [72], and some human therapeutic targets do not exist in animal hosts. For these reasons, the practicability of animal models in evaluating drug sensitivity of gliomas is greatly reduced. Glioma organoids induced by GBM, especially those produced from patient-derived cells, provide an effective platform for drug screening, because it perfectly overcomes the two major problems mentioned above.

The glioma organoids biobank has three features that make it an attractive platform for GBM drug testing. First of all, the rapid generation of glioma organoids makes it possible to test the drug response before clinical treatment, thus achieving a truly personalized drug treatment. Secondly, a summary of many aspects of GBM heterogeneity in a single glioma organoids shows that they may simulate drug reactions better than traditional GBM models. Third, the size and diversity of the glioma organoids biobank, as well as its relative ease of further expansion, pave the way for understanding the relationship between glioma organoids genotypes and cell states in response to drugs [73].

In 2020, Zhang LY et al. reported a real-time integrated system by generating 3D ex vivo cerebral organoids and in vivo xenograft tumors based on glioma patient-derived tissues and cells. The system faithfully recapitulated the histological features, response to chemotherapy drugs, and clinical progression of their corresponding parental tumors. In conclusion, they developed an integrated system of parallel models from patient-derived glioma cerebral organoids and xenografts for understanding the glioma biology and prediction of response to chemotherapy drugs, which might lead to a new strategy for personalized treatment for this deadly disease [74].

### For personalized cancer treatment

Glioma organoids can not only be used for drug sensitivity test, but also can be used for personalized treatment of patients. Because with the emergence of target capture sequencing technology used to identify genomic changes, coupled with the fact that patient-derived organ-like cultures can persist in real-time in vitro drug testing of genomic representative cells, the technology to provide personalized treatment has become possible [75].

In 2020, Loong HH and colleagues reported a case report that used organoids technology to provide personalized treatment for patients. GSC is extracted from the intraoperative tissue of patients undergoing the first operation, and then glioma organoids are cultured using a method similar to Rich [7]. The organoid derived from the patients are then sequenced, which provides information for subsequent drug candidates. Through sequencing, the authors found that two frameshift indels of NF1 shared across all samples, together with a highly consistent profile of copy number alterations, accentuated on a shared clonal origin. Heterozygous PTEN copy loss along with PTENW111* nonsense mutation in the initial tumor suggested a bi-allelic loss of PTEN function that likely induced mTOR signaling. Meanwhile, intratumoral heterogeneity at first clinical presentation was evident through low frequency of hotspot PIK3CAH1047Q mutations detected in the initial tumor but enriched at relapse. Together, these genetic abnormalities underscored activation of the PI3K/ AKT/mTOR pathway. Next, the authors tested a panel of genome-guided drug candidates on cultured patient-derived organoid to predict drug sensitivity. The authors found that the patient's TMZ resistance reappeared in patient-derived organoid. So they further tested FDA-approved anti-cancer drugs associated with either PTEN loss/PTENW111* such as mTOR inhibitor everolimus or NF1 frameshift such as MEK inhibitor cobimetinib. Compared with other drugs, patient-derived organoid is more sensitive to everolimus cytotoxicity, so everolimus is chosen as the candidate drug. The patient was initially started on everolimus 5 mg daily which was further stepped up to 10 mg daily. Reassessment imaging after four weeks of treatment showed the residual enhancing tumor to be less bulky with partial relief of mass effect on the right lateral and third ventricles, and midline return of structures. This illustrates the strong dependency of this tumor on the PTEN pathway for growth. Identification of this dependency has revealed an actionable target for personalized treatment [76].

The author of the above case extracted glioma stem cells from the tumor tissues of glioma patients, and then constructed glioma organoids by a method similar to Rich [69]. Then through genome sequencing to observe the gene mutations of this patient with glioma, so as to conduct corresponding drug susceptibility experiments for specific gene mutations. In the end, patients get the greatest benefit. Therefore, the essence of the so-called individualized accurate tumor data is to provide personalized treatment options by identifying and targeting the genomic and molecular aberrations of individual patients' tumors. Compared with traditional long-term cultured cancer cell line models, the advantage of glioma organoids is that they can more accurately summarize the molecular characteristics and biology of the disease. Compared with the PDX model, the glioma organoids shorten the modeling experiment and also reduce the cost of the model [77–79]. More importantly, chemical screening using glioma organoids has significant advantages over PDX because it greatly increases the number of chemicals that can be used in multiple doses, which is necessary to produce reliable drug response parameters. Glioma organoids represent the unique biology of each corresponding tumor and provide a more accurate model system for evaluating drug response.

### Used to explore glioma and its TME

Cancer is not a cellular autonomous disease, but a disease in which cancer cells are closely related to the biology of host cells. This is especially true for GBM, which does not metastasize, but spreads and eventually kills the patient by spreading and infiltrating into the surrounding normal brain tissue. Therefore, TME plays a key role in studying the heterogeneity, plasticity and evolution of gliomas [80]. Glioma CSCs can not only renew [81], proliferate [82] and separate into different tumor cells, but also interact with different tumor components such as ECM, intercellular (tumor-associated fibroblasts, immune cells, differentiated nerve cells, etc.) and even blood–brain barrier (BBB) through tumor-derived pericytes. So as to establish a good ecological environment for further malignant transformation and treatment of drug resistance [83]. Therefore, the study of TME is particularly important.

However, traditional 2D culture can not simulate this vital cell–cell interaction and tumor microenvironment. Although the patient-derived mouse xenotransplantation model solves the problem of host-tumor cell interaction, the model is not perfect due to significant species differences in gross neuroanatomy (e.g., underdeveloped murine neocortex) and cellular level (e.g., astrocytic dendritic complexity and transmission speed of calcium transients in murine versus human astrocytes) [84]. The Glioma Organoids model addresses many of the limitations of previous models because it allows people to study patient-specific GBM in vitro in a microenvironment similar to the primitive human brain. The biological behavior and histopathological characteristics of GBM derived from patients growing in brain organs are closely related to surgical and autopsy specimens, which proves the clinical correlation of this model. This was further confirmed by the maintenance of patient-specific EGFR amplification and phosphorylated RTK signals by glioma organoids, as well as the spontaneous formation of Glioma Organoids microtubules, and microstructure features were also found in situ.

### Construct patient-derived orthotropic xenografts

Patient-derived xenografts (PDXs) represent a mature preclinical cancer model that allows the propagation and study of human tumors in immunodeficient mice. Usually, PDXs models are obtained by subcutaneously or in situ implantation of patient tumor tissue fragments, but for glioma, the success rate of modeling is about 50% [85]. Moreover, implanting tumor fragments directly into the brain is technically challenging and may lead to unreplicable tumor growth. The patient-derived orthotopic xenograft (PDOXs) established by implanting patient-derived glioma organoids into the brain ensure technical feasibility and standardization, while avoiding the selection and adaptation of glioma, and can better generalize The histopathological characteristics and TME of the tumor are described. Different culture models of glioma organoids will produce tumors during xenotransplantation in the brain, and can well summarize the histopathological characteristics of glioma patients, such as invasion and angiogenesis. Not only untreated glioma organoids can develop and cause tumors in the body, but also treated glioma organoids can also develop and cause tumors in the body. This model can be used to study tumor changes before and after treatment. It is also possible to generate paired longitudinal models from tumor samples collected from the same patient at different time points, thereby reproducing the progression of the disease over time [86]. This model is a valuable tool for studying tumor evolution and treatment resistance in a personalized in vivo environment. Not only that, genetically engineered glioma organoids have also been shown to cause intracranial tumors in the body [58].

PDOXs allow tumor substances in the body to multiply in sufficient brain microenvironment, including structures (vasculature, blood–brain barrier), cells (neurons, glial, microglia/macrophages) and metabolic components (cerebrospinal fluid, Interstitial fluid). This method can also avoid the loss of TME, additional aberrations and cell state selection due to long-term culture and expansion of organoids in vitro. Because glioma organoids can be further obtained from the established PDOX and continuously transplanted to maintain the patient's tumor for multiple generations [86]. Moreover, researchers have shown that PDOXs can remain stable in mice for several generations. The applications of PDOXs range from in vivo drug validation studies, optimization of magnetic resonance imaging protocols, dynamic analysis of tumor metabolism in vivo using isotope tracers, genetic and phenotypic analysis, to identification of new biomarkers and therapeutic targets. Therefore, this kind of PDOXs represents an invaluable patient "incarnation" for downstream experimental needs and applications.

## Challenges of cerebral organoids in glioma applications

Although the use of glioma organoids has brought new opportunities for the diagnosis and treatment of gliomas, there are also several major challenges. Below we will list several current difficulties and their corresponding solutions.

### The maturation of cerebral organoids is too low

Some studies have shown that the maturity of brain organoids produced in vitro is only equivalent to that of the brain at 8–10 weeks of pregnancy [52, 87]. However, gliomas usually occur in the adult brain. Because Goranci-BuzhalaG and colleagues have found that glioma stem cells are better at invading the mature brain [88]. In detail, Goranci-BuzhalaG et al. comparing GSC integrations occurring in 20-, 40-, and 60-day-old organoids revealed that the integration behavior of patient-derived GSCs is inversely correlated with the organoids’ age. This is because mature organoids could provide suitable microenvironmental determinants for GSC growth, an aspect that is consistent with the fact that neuronal activity generates mitogenic factors promoting glioma growth [89, 90]. They therefore suspected that the relative slow integration of GSCs in 20-day-old organoids might be due to the lack of sufficient mitogenic factors secreted by neurons. Indeed, exogenously supplementing mitogenic factors Neuroligin-3 to 20-day-old organoids promoted GSCs integration. Therefore, the production of mature brain-like organs is very important for the study of gliomas.

But surprisingly, Liu SJ and his colleagues generated “mature” human brain organoids (MBOs) that more closely reflect the differentiated cellular state of the postnatal human brain [41]. Because astrocytes are the most abundant cell types in the adult brain [91]. So they used a scheme to produce purebred mature human astrocytes from human iPSCs (iAstrocytes). Using an isogenic iPSC (WTC11) clone that carries an inducible Neurogenin2 (NGN2) transgene, they also generated homogenous cultures of mature cortical neurons (i3Neurons) with NGN2 induction [92, 93]. MBOs can be formed from iAstrocytes and i3Neurons by mixing and co-culture of these cell types in defined numbers and ratios (from a 1:1 ratio to solely iAstrocytes or i3Neurons) [94].

As a 3D tissue platform for the study of human glioma, MBOs offer certain characteristics that distinguish them from embryonic brain organoids and GBM-derived tumor organoids. In contrast to embryonic brain organoids that mimic early stages of fetal brain development, MBOs are assembled from cell populations that are more mature and postmitotic [92, 94]. As a result, they better reflect the state of the mature brain.

### Lack of complete tumor microenvironment

Although the MBO model solves the problem of the maturity of brain organs, it also has some defects. One limitation of MBO-glioma model is the absence of a complete tumor microenvironment, which includes microglia, stromal cells, and tumor-infiltrating lymphocytes, among other cell types [95, 96]. Glioma is a late-onset disorder, and thus more meaningful glioma modeling requires brain organoids harboring mature cell types of astrocytes, oligodendrocytes, myelinated neurons, and immune defense cell types of microglia. Glioma microenvironment contains a number of cell types as another component of the tumor dynamics. These cells actively interact with glioma cells [13]. Therefore, how to simulate the complete tumor microenvironment has also become a thorny problem for glioma organs. However, scientists are also working in this direction.

To date, several protocols that generate brain organoids allow the differentiation of astrocytes, mature neuronal cell types, and even surprisingly microglia-like cells [52, 97]. Microglia are essential cell types as glioma cells communicate with them via releasing extracellular vesicles [98, 99]. Microglial cells in brain organoids are unexpected, as microglial cells do not originate from neuroectoderm, which is the primary germline to generate neural lineages. Dual-SMAD inhibition is a mechanism that can trigger the neuroectoderm formation. Ormel et al. took a thoughtful approach of generating brain organoids omitting SMAD inhibitors, which surprisingly developed Iba-1-positive microglial cells with their characteristics of ramified morphology [100]. Omitting retinoic acid at the initial stage of differentiation condition, Ramani et al. have also observed microglial cells and astrocytes in their organoids [101]. It is hoped that more results can be achieved in the near future.

### Lack of vascular network formed by endothelial cells

Another critical missing factor in the brain organoids is endothelial cells. The formation of blood vessels is very important for organoids. Because when an organoids grows more than a certain size without forming a vascular network, it becomes a problem for nutrients to spread to cells in organoids [102]. Because cells more than 200–400 μ m from the cell surface are unable to absorb enough oxygen and nutrients through separate diffusion, the center of the organoid may be necrotic [103]. In addition, the angiogenesis of brain tissue is a key factor in the development of brain tissue [104], and GSCs usually exists around blood vessels [105]. Therefore, in order to correctly summarize the brain tissue for the purpose of disease model, the vascularization of brain organs is necessary.

Excitingly, several methods for inducing blood vessel formation on brain organs in vitro have recently been developed. One method is to express ETV2 in genetically modified human pluripotent stem cells. Over time, this leads to the formation of blood vessel-like structures in the final organoids [102]. Another method is to embed human endothelial cells in Matrigel to which the early stage organoids are added. Over time, this causes human endothelial cells to self-assemble into capillaries on the periphery of organoids and invade the vascular network [103]. Although in the above schemes, obvious blood vessel network formation can be found on the periphery of organoids, the formation of blood vessel network towards the center of organoids is less. Therefore, in vitro technology promotes the formation of functional vascular networks, but it cannot fully penetrate the entire brain organoids.

Another method of vascularization is to transplant organoids into immunodeficiency mouse models. For organoids that have not been treated to promote angiogenesis in vitro, it has been proved that for brain organoids grown for 40–50 days, rat blood vessels begin to invade 7–10 days after implantation. The human-specific CD31-labeled immunostaining is inconsistent with the newly formed vascular network, indicating that it is of host origin. However, unlike in vitro vascularization efforts, the vascular network is not limited to the periphery, but penetrates the entire organoid [106, 107].

## Conclusion

Although there are a variety of challenges in the study of glioma organs, but with the efforts of generations of researchers, they will eventually be easily solved. In addition, new emerging technologies, such as 4D real imaging technology [108], microfluidic technology [109], organ-on-chip technology [110] and single-cell sequencing technology [51], will surely bring to glioma organoids New insights reveal the untapped potential of these models. In general, GB organoids have aroused a lot of hope, and their potential will grow further in the near future, which will eventually lead to personalized treatments for glioblastoma.

## Data Availability

Not applicable.

## Abbreviations

WHO: World Health Organisation
GBM: Glioblastoma multiforme
CNS: Central nervous system
GSCs: Glioma cancer stem cells
EGF: Epidermal growth factor
FGF2: Fibroblast growth factor
TME: Tumor microenvironment
PDXs: Patient-derived xenografts
GEMMs: Genetically engineered mouse models
iPSC: Induced pluripotent stem cells
EGFR: Epidermal growth factor receptor
IDH: Isocitrate dehydrogenase
TERT: Telomerase reverse transcriptase
PTEN: Phosphatase tensin homologue
NF1: Neurofibromatosis type 1
PDGFRα: Platelet-derived growth factor receptor alpha
TP53: Tumor protein p53
RB: Retinoblastoma protein
CDKN2A: Cyclin-dependent kinase inhibitor 2A
MGMT: Methylation of O6-methylguanine-DNA methyltransferase
SB: Sleeping beauty
neoCOR: Neoplastic brain organs
ECM: Extracellular matrix
GLICO: Cerebral organoid glioma
BBB: Blood–brain barrier
MBOs: Human brain organoids
iAstrocytes: Mature human astrocytes from human iPSCs
NGN2: Neurogenin2
i3Neurons: Mature cortical neurons
